# Clinical trial of a method for confirming the effects of spinal anesthesia in patients with spinal cord injury

**DOI:** 10.1007/s00540-012-1429-z

**Published:** 2012-06-19

**Authors:** Akiko Takatsuki, Masahide Ohtsuka

**Affiliations:** 1 Department of Anesthesia, Kanagawa Rehabilitation Hospital, 516 Nanasawa, Atsugi, Kanagawa 254-0121 Japan; 2 Department of Intensive Care Medicine, Yokohama City University Medical Center, 4-57 Urahune-cho, Minami-ku, Yokohama, Kanagawa 232-0024 Japan

**Keywords:** Spinal cord injury, Spinal anesthesia, Spasticity

## Abstract

In this case series study, we prospectively examined whether it might be possible to check the effect of spinal anesthesia (SA), based on the disappearance of lower extremity reflexes and spasticity, in patients with spinal cord injury (SCI), in whom the effect cannot be confirmed by the pinprick test or by using the Bromage scale. In 40 patients with chronic, clinically complete cervical SCI who were scheduled to receive SA, pre-anesthetic examination revealed that the Babinski sign, patellar tendon reflex, and spasticity (assessed using the Ashworth scale) were all positive in 31 patients, while two of these three pre-anesthetic assessment parameters were positive in eight patients. The effect of SA in these 39 patients (97.5 %) was confirmed by demonstrating the absence of both the Babinski sign and patellar tendon reflex and loss of spasticity after SA. Our results suggested that the effect of SA can be confirmed by the disappearance of the Babinski sign and patellar tendon reflex and loss of spasticity in most patients with complete cervical SCI, although determination of the level of the block is difficult. In conclusion, loss of the Babinski sign, patellar tendon reflex, and spasticity might be useful for checking the effect of SA in cervical SCI patients.

## Introduction

Anesthesia for patients with chronic spinal cord injury (SCI) is required for the prevention of autonomic dysreflexia and spasticity [1]. Spinal anesthesia (SA) is an effective anesthetic method that can prevent autonomic dysreflexia and spasticity by blocking afferent nerve impulses [1, 2]. It has been pointed out, however, that it may be impossible to determine the level of the block in SCI patients [1].

We had experienced a few SCI patients in whom autonomic dysreflexia developed intraoperatively following SA with only needle aspiration of cerebrospinal fluid. Therefore, we attempted to establish a method of clinically confirming anesthetic effects based on changes in neurological findings before the start of surgery.

A previous case report described loss of lower extremity spasticity in a patient with cervical SCI following SA, although the level of the block could not be determined [3]. Although disappearance of spasticity and the patellar tendon reflex (PTR) is known to occur following SA in patients with cervical SCI [1, 4, 5], it still remains unclear whether all patients with cervical SCI show lower extremity spasticity and positive PTR before anesthesia. Therefore, to clarify this problem, we examined patients with chronic cervical SCI who demonstrated spasticity (determined using the Ashworth scale), and had a positive PTR and the Babinski sign (BS) before anesthesia. We then assessed the patients for spasticity and the presence of PTR and BS post-anesthesia and on postoperative day 1, in order to confirm the effects of and recovery from SA.

## Case series

The study population comprised patients with chronic, clinically complete cervical SCI who received SA between April 2010 and August 2011 at Kanagawa Rehabilitation Hospital. The protocol of the study was approved by the Ethics Committee of this hospital and written informed consent was obtained from all patients. Patients with the usual contraindications to SA (coagulopathy, local infection of the puncture site, patient refusal) were excluded from this study.

Before anesthesia and at 10 and 15 min after SA, patients were tested for the presence of PTR and BS, while flexion/extension at the hip joints, knee joints, and ankle joints was assessed using the Ashworth scale for spasticity grading (Table 1) [6]. Patients were assessed as positive for spasticity when any of these joints showed grade 1, 2, 3, or 4 spasticity on the Ashworth scale, and as negative when all these joints demonstrated grade 0 spasticity. Muscle spasms and the onset of clonus were also regarded as positive findings for PTR/BS and spasticity when the reflexes and spasticity were examined. The above testing/grading was performed by the same anesthesiologist in all patients.

**Table 1:** Ashworth scale

0: No increase in tone
1: Slight increase in tone giving a “catch” when the limb is moved in flexion or extension
2: More marked increase in tone, but limb easily flexed
3: Considerable increase in tone–passive movement difficult
4: Limb rigid in flexion as well as extension

In the patients who underwent urological surgery, changes in rectus abdominis muscle tone were assessed by palpation before and after SA. For SA, 0.5 % hyperbaric bupivacaine in 7.3 % glucose was administered via the L3–4 or L4–5 intervertebral space with the patient in the lateral position. If the patient was assessed as being positive for either PTR, BS, or spasticity at 15 min after SA, SA was re-administered and surgery was allowed to commence only after disappearance of PTR/BS and spasticity bilaterally. On postoperative day 1, patients were re-examined for the presence of PTR/BS and spasticity, using the Ashworth scale, to compare the results with the preoperative data.

In all 40 patients enrolled in the study, surgery could be performed without the need for general anesthesia and without spasticity, severe hypotension, autonomic dysreflexia (defined as indirect systolic blood pressure of 160 mmHg or more in response to surgical stress), or any other serious complications. In all cases, the surgeons commented that they were able to perform the intraoperative procedures without problems. Patient characteristics, hemodynamic data, and pre-anesthetic neurological findings are shown in Table 2.

**Table 2:** Patient characteristics, hemodynamic data, and pre-anesthetic positive rates for Babinski sign, patellar tendon reflex, and spasticity (using the Ashworth scale)

Age (years)	44 (16–69)
Weight (kg)	57 ± 14 (35–91)
Height (cm)	168 ± 8 (150–181)
Male/female	30/10
Years post-injury	10 (0.5–45)
Cause of injury	
Traumatic/non-traumatic	37/3
Injury level	
C4/C5/C6/C7/C8	8/8/20/2/2
Type of surgery	
Urological surgery	23 (57.5 %)
Decubitus ulcer surgery	11 (27.5 %)
Lower extremity surgery	2 (5 %)
Hemorrhoid surgery	4 (10 %)
Dose of 0.5 % hyperbaric bupivacaine for spinal anesthesia (mg)	10 (8–15)
Incidence of hypotension (SBP <80 mmHg)	4 (10 %)
Lowest SBP within 30 min after SA (mmHg)	96 ± 17 (56–142)
Lowest HR within 30 min after SA (beats/min)	69 ± 11 (46–92)
Ephedrine supplement	4 (10 %)
Dose of ephedrine (mg)	4 (4–20)
Pre-anesthetic positive neurological findings	
Babinski sign	38 (95 %)
Patellar tendon reflex	35 (87.5 %)
Spasticity	36 (90 %)
All three parameters positive	31 (77.5 %)
Positive for any two of the three parameters	8 (20 %)
Positive for any one of the three parameters	0 (0 %)
All parameters negative	1 (2.5 %)

Data are presented as medians (ranges), means ± standard deviation (ranges), or numbers (%)

Ephedrine in increments of 4 mg was given intravenously to treat hypotension, defined as decrease in SBP <80 mmHg

*SBP* systolic blood pressure, *HR* heart rate, *SA* spinal anesthesia

In terms of the pre-anesthetic data, 31 of the 40 patients showed positive PTR, BS, and spasticity, using the Ashworth scale, while eight patients were positive for two of these three parameters (Table 2). The effects of SA in these 39 patients were confirmed by demonstrating the disappearance of PTR/BS and spasticity after SA. In the remaining patient, who had a history of C6 SCI sustained 34 years previously and T11-12 vertebral fracture dislocations sustained 4 years before the present operation, the effect of SA could not be confirmed due to the absence of PTR/BS and spasticity pre-anesthesia.

Grading of spasticity on the Ashworth scale was not possible in five patients because of contracture of the ankle joints, although these patients were found to be positive for spasticity in the other joints. In another patient, the patellar reflex and spasticity grading on the Ashworth scale at the knee joint could not be assessed due to fracture of the patella; however, PTR/BS and spasticity were positive in the remaining joints and in the contralateral lower extremity.

In two patients who showed unilateral positive PTR/BS after SA, re-administration of SA, with the patient in the lateral position with the positive side down, resulted in loss of the PTR/BS bilaterally.

By the time of their discharge from the operating room (30–180 min after anesthesia), PTR/BS and spasticity had disappeared in all patients. Although 19 of the 41 patients perceived the disappearance of anesthetic effects due to the perception of spasticity and the onset of autonomic dysreflexia 4–8 h after SA, the other patients did not have such a perception. However, the neurological findings on postoperative day 1 were consistent with those observed prior to anesthesia in all patients. The patients told us that their lower limb sensations were the same as those before anesthesia.

## Discussion

In this study, the effect of SA was confirmed in 39 of 40 patients (97.5 %) by demonstrating the absence of the assessment parameters of PTR, BS, and spasticity after SA. Our results suggest that the effect of SA in most patients with complete cervical SCI can be confirmed by the disappearance of PTR, BS, and spasticity.

Spasticity is a sign of upper motor neuron syndrome [7], which results from corticospinal tract damage. The syndrome is associated with signs such as spasms, spastic paralysis, hyperactive tendon reflexes, and BS, and is thought to result from adaptive changes in transmission in the spinal networks distal to lesions of descending motor pathways [8, 9]. The BS is thought to be part of the general withdrawal reflex synergy released by a lesion of the supraspinal pathways that project onto the interneuronal zone of the lumbosacral cord (L5, S1) [10]. The PTR is a spinal reflex, with its center located in the lumbar spinal cord (L2–4), and the reflex is often enhanced by damage to the upper motor neurons.

Most patients in the present study demonstrated spasticity, determined using the Ashworth scale, and positive PTR/BS before SA, because, as it was inferred, most patients with chronic cervical SCI, such as our patients, present with signs of upper motor neuron syndrome.

However, some patients in the present series exhibited negative PTR and BS even before SA. Although the cause for these findings remains unclear, they can probably be explained by flaccid paralysis caused by peripheral neuropathy [11].

There were eight patients who were negative for any one of the three assessment parameters employed in this study, i.e., spasticity using the Ashworth scale, PTR, and BS. Testing all of the three assessment parameters might, therefore, increase the probability of confirmation of the effect of SA.

One patient in this study was found to be negative for all three of the assessment parameters before SA; he had developed loss of spasticity after suffering T11–12 vertebral fracture dislocations 4 years earlier. It is likely that this patient had developed flaccid paralysis due to a lower spinal cord injury associated with the T11–12 fracture dislocations.

Two patients in this study showed unilateral positive PTR and BS after the first SA, with bilaterally negative findings after SA was performed again. It is thus thought that the effect of SA might be checked by ascertaining whether positive PTR/BS becomes negative following SA. The return of PTR, BS, and spasticity on the Ashworth scale to pre-anesthetic levels on postoperative day 1 in all the patients studied indicated the disappearance of the effects of SA by the following day.

Nevertheless, determination of the level of the block in SCI patients is difficult. It is only possible to indirectly estimate the effectiveness of anesthesia from the absence of BS (S1–L5), PTR (L2–4), or hip joint relaxation on the Ashworth scale (L1). The level of the block required for surgeries for hemorrhoids, decubitus ulcers of the sacral or ischial region, and for the lower extremities is L1 or lower. These surgeries can be smoothly performed without any intraoperative autonomic dysreflexia or spasticity after confirming that PTR/BS and spasticity have become negative. Using these assessment parameters alone, however, it is difficult to confirm the adequacy of the level of SA in patients for urological surgeries, such as cystostomy and transurethral operations, in which block levels of T10 or higher are required [12]. Interestingly, among the 23 patients in this series who underwent urological surgeries, there were two patients in whom relaxation of rectus abdominis muscle tone could be confirmed by palpation. Hence, it is possible that changes in rectus abdominis muscle tone might be useful for checking the effect of SA in some SCI patients.
